# Understanding Aotearoa New Zealand University Students Intentions to Seek Help If Experiencing Mental Distress: A Comparison of Naturalistic and Interventional Findings

**DOI:** 10.3390/ijerph192315836

**Published:** 2022-11-28

**Authors:** Andre Mason, Grace Johnstone, Benjamin C. Riordan, Celia Lie, Charlene Rapsey, Gareth J. Treharne, Kyungho Jang, Sunny C. Collings, Damian Scarf

**Affiliations:** 1 Department of Psychology, University of Otago, P.O. Box 56, Dunedin 9054, New Zealand; 2 Centre for Alcohol Policy Research, La Trobe University, Melbourne, VIC 3086, Australia; 3 Department of Psychological Medicine, University of Otago, Dunedin 9016, New Zealand; 4 Faculty of Health, Victoria University, Wellington 6012, New Zealand

**Keywords:** mental distress, help-seeking barriers, suicidal ideation, university students

## Abstract

University students globally are consistently identified as a vulnerable group for mental distress and suicide. Despite this, students report low engagement in help-seeking behaviours. This series of studies aimed to assess barriers to help-seeking for students and the impact of an intervention that sought to increase support-seeking intentions. In Study 1, 373 undergraduate psychology students completed items related to depression, anxiety, suicidal ideation, stigma, and help-seeking intentions. In Study 2, 133 undergraduate psychology students were randomly allocated into one of three intervention groups (control, infographic, video) and completed measures as used in Study 1. Despite experiencing clinically relevant symptoms and recent suicidal ideation, students in Study 1 tended to report low intentionality to seek help, citing perceptions that their distress was not serious enough or a desire to handle their issues independently. In Study 2, an infographic about different support services increased student’s intentions to access support services and reduced their perception that their issues were not serious enough. Overall, Aotearoa New Zealand students endorsed similar barriers to help-seeking as students in other countries. Importantly, we demonstrated that a simple infographic intervention reduced perceptions regarding these common barriers and may increase students’ knowledge about when to seek help.

## 1. Introduction

Recent data from the World Health Organization (WHO) World Mental Health Surveys International College Student Project has provided important insights into students’ attitudes towards help-seeking and the barriers they perceive to seeking treatment. Of particular concern, first-year university students worldwide have low engagement with support services [1] and are hesitant to seek support for emotional distress, including suicidal ideation [2]. For example, in a large cross-national survey (*n* = 9939), only 24.6% of students reported that they would definitely seek support for mental distress, with many citing reasons such as a *“preference to handle the problem alone”* and a desire to *“talk with friends or relatives instead”* [2]. These low levels of engagement with support services are coupled with consistent evidence that student populations are a vulnerable group for mental distress and suicide, with estimates that approximately 34.9% of first-year students struggle with at least one mental disorder [3], and 17.2% experience suicidal ideation [4].

Beyond cross-sectional, descriptive analyses, it is important to consider (a) longitudinal designs that test whether student’s help-seeking attitudes and behaviours naturally shift over time and (b) experimental designs that test interventions developed to positively shift student’s attitudes and behaviours towards seeking help. To date, while intervention studies have shown promise at increasing mental health literacy and positive shifts in attitudes towards help-seeking [5,6], they have had limited effect on increasing the likelihood of engagement in help-seeking behaviours [5]. For example, one recent study developed a mental health promotion video and brochure which aimed to increase mental health knowledge and attitudes toward help-seeking among students [5]. The video and brochure provided information on mental illness, strategies for self-help, and encouraged help-seeking when experiencing distress. While the video and brochure increased mental health knowledge, there was little evidence it influenced help-seeking behaviour. Given this, more work is needed to develop mental-health promotion tools that not only increase mental health knowledge but also have a clear impact on help-seeking for students.

## 2. The Current Studies

The aim of the current studies was to investigate barriers to help-seeking in students in Aotearoa New Zealand. In Study 1, we assessed barriers to help-seeking and, extending previous research, used a longitudinal design to test for changes to barriers to help-seeking over a one-year period. In Study 2, we trialled two approaches to reduce these barriers to help-seeking using a short mental-health promotion video and infographic that briefly covers the main support services available locally to university students.

## 3. Study 1

### 3.1. Method

#### 3.1.1. Participants

Participants were 373 undergraduate psychology students enrolled at a large, campus-based university in Aotearoa New Zealand. Students were relatively evenly split between their first (*n* = 207) and second (*n* = 166) year of study. Participants were between the ages of 18–25 years old (Mean (M) = 19.59, Standard Deviation (SD) = 1.18) and predominantly identified as female (*n* = 313; 60 males, one non-binary). The majority of the participants self-identified as New Zealand European only (75.87%; *n* = 283), followed by Māori (*n* = 21), Pasifika (*n* = 8), and Asian (*n* = 38). Twenty-one (5.63%) stated specific ethnicities which generally fell into other European regions meaning 79.09% were White.

One hundred and eighty-one participants (48.5%) completed the follow-up survey. Again, the majority were female (*n* = 155, 85.6%; *n* = 25 male, *n =* 1 non-binary) and identified as New Zealand European only (*n =* 144, 79%), followed by Māori (*n* = 12), Pasifika (*n* = 3), and Asian (*n* = 16). Four (2.21%) stated specific ethnicities which generally fell into other European regions. Finally, 92 (50.8%) were now in their second year while 89 (49.2%) were in their third year.

#### 3.1.2. Procedure

This study was approved by the host university Ethics Committee (H20/029). Undergraduate students taking a first- or second-year psychology paper were invited to participate for course credit related to learning about research; students take undergraduate psychology papers from several majors across science and arts. Students who expressed interest in participating were emailed about the study and, if they chose to participate, were asked to complete a brief online survey through Qualtrics. Electronic consent was obtained, and every student was given the contact details for the research team, the student health facility, and emergency psychiatric services, in addition to a list of other support services and telephone helplines. A second follow-up survey was sent to students approximately one year later which contained the same items and information.

#### 3.1.3. Measures

*Demographic.* Participants completed a questionnaire on general demographic information such as age, ethnicity, and gender.

*Suicidal Ideation.* The item “thoughts that you would be better off dead or hurting yourself in some way” from the Patient Health Questionnaire-9 (PHQ-9) was used as a proxy measure for recent suicidal ideation. Participants respond to the frequency of these thoughts using a four-point Likert scale ranging from “not at all” to “nearly every day” (*Cronbach’s alpha (α)*: Time 1 = 0.84, Time 2 = 0.89). This item of the PHQ-9 has previously been used as an indicator of suicidal thoughts [7] and shown to reliably predict an increased risk of suicide attempt or death [8,9].

*Depression, Anxiety, Stress.* The Depression, Anxiety, and Stress Scale (DASS-21) is a 21-item questionnaire with seven items assessing each of the three mental health aspects: depression (*Cronbach’s alpha (α)*: Time 1 = 0.89, Time 2 = 0.90), anxiety (*α*: Time 1 = 0.86, Time 2 = 0.85), and stress (*α*: Time 1 = 0.84, Time 2 = 0.84). Participant responses were recorded using a four-point scale from 0 (never) to 3 (almost always).

*Stigma.* The five-item Stigma Scale for Receiving Professional Psychological Help (SSRPH) was used to measure public stigma associated with seeking support (e.g., “seeing a psychologist for emotional or interpersonal problems carries social stigma”). Participants rated their level of agreement/disagreement with each statement using a four-point Likert scale ranging from “strongly disagree” to “strongly agree” (*α:* Time 1 = 0.74, Time 2 = 0.86). Higher levels indicated a greater level of perceived stigma associated with seeking professional psychological help.

*Help-Seeking Barriers.* Barriers to help-seeking were asked with respect to accessing the University’s subsidised primary care health service and non-university mental health services. Specifically, using a five-point Likert scale ranging from “definitely would go” to “definitely would not go”, participants were first asked to respond to two questions adapted from Bruffaerts et al. [10]: “*As you might know, [the university’s health service] offers counselling and psychological services to help students who have emotional problems. If during this year you developed a problem that caused you a lot of distress and interfered with your work, how likely would you be to go to [the university’s health service]?*” and “*How likely would you be to go somewhere else for help, like to your doctor, a mental health professional or religious advisor?*”. A third question, “*If you decided NOT to seek help if you developed such a problem, how important do you think each of these would be as reasons for NOT seeking help?*” was then asked to all students that did not indicate that they definitely would seek help. Students were asked to indicate how much they agreed with ten possible reasons (e.g., “you would want to handle the problem on your own”) using a five-point Likert scale ranging from “unimportant” to “very important”, with higher scores indicating stronger levels of agreeance.

#### 3.1.4. Analyses

All analyses were conducted using R (version 4.2.1 [11]). First, descriptive statistics and frequencies were calculated (package: psych [12]), and Spearman’s rho correlations were used to determine the relationship between clinical characteristics (e.g., suicidal ideation, depressive symptoms) and the perceived help-seeking barriers, and between clinical characteristics themselves. Wilcoxon rank sum tests (package: ggplot2 [13], Base R [11]) were then used to determine whether help-seeking intentionality differed by gender or study level and, after filtering out responses of “Definitely would go” (as these would be unexpected to lead to the endorsement of perceived barriers), whether the perceived barriers to help-seeking differed between gender or year level.

Wilcoxon signed-rank tests were then used to determine whether a student’s intentions to seek help when they were in distress, or their perceived barriers to seeking help, had naturally shifted over time. Finally, linear mixed models were used to determine whether students’ likelihood to seek help could be predicted by the perceived barriers or any barrier X time interactions. Since only one person identified as non-binary, comparisons between gender were limited to comparisons between female and male participants.

### 3.2. Results

#### 3.2.1. Clinical Characteristics

Using the clinical cut-off scores for the DASS-21 [14], 59.9% of the sample scored within the normal range for anxiety, 15.2% in the mild range, 9.8% in the moderate range, 7.7% in the severe range, and 10.4% in the extremely severe range at Time 1. For stress, 69.8% of the sample scored within the normal range, 12.7% in the mild range, 11.1% in the moderate range, 4.8% in the severe range, and 1.6% in the extremely severe range. Additionally, 58.7% of the sample scored within the normal range for depression, 14.5% within the mild range, 14.3% within the moderate range, 6.8% within the severe, and 5.7% within the extremely severe range. No clinically significant changes were observed between Time 1 and Time 2 for depression, anxiety, or stress. A further 16.5% of the sample (*n* = 73) reported having suicidal ideation for at least several days in the past two weeks.

The relatedness between these characteristics and the perceived barriers to help-seeking can be found in Table 1. Briefly, at Time 1, the frequency of suicidal ideation was associated with greater endorsement of concerns about time, scheduling, or transportation, and fears about the impact on their professional careers, yet reduced endorsement of being willing to talk to friends or family. Symptoms of depression, anxiety, and stress were associated with concerns about the effectiveness of potential treatments, embarrassment, a preference to talk to friends or family, problems with time, scheduling, and transport, fears about the impact on their careers, and worry that people would treat them differently. The same relationships were found with stigma, with the exception of concerns related to time, scheduling and transport, which was not related to stigma.

These relationships largely remained consistent at Time 2 for suicidal ideation, depression, and stigma. One exception was the belief that their distress was not serious enough for professional support; this belief was positively associated with these characteristics. At Time 2, anxiety and stress were no longer related to perceived embarrassment or worries that they would be treated differently. Anxiety was further unrelated to concerns about treatment effectiveness, time, scheduling, or transport, or fears of the impact on career.

#### 3.2.2. Cross-Sectional Analyses

*Help-Seeking Behaviours.* Only 15.0% of students reported that they would “definitely go” to the university’s health service if they developed a problem that caused emotional distress or interfered with their work, with a further 33.16% saying they “probably would go” (see Table 2). Responses did not differ by gender, *W* = 8250, *p* = 0.12 or year level, *W* = 17,248, *p* = 0.95. Additionally, only 14.71% of students reported that they “definitely would go” to another professional for help, with 31.28% saying they “probably would go.” Female participants were more likely to seek help from another support service, compared to male participants, *W* = 7246, *p* < 0.01. No differences were observed across year groups, *W* = 17,081, *p* = 0.92.

*Stigma.* Overall, participants tended to disagree that seeking professional psychological support is associated with stigma (M = 9.470, SD = 2.72). However, male participants indicated greater perceived stigma than female participants, *W =* 11,614, *p* < 0.001. No differences in perceived stigma were observed between year groups, *W* = 18,603, *p =* 0.17.

*Reasons for not seeking help.* For participants who did not respond with “definitely would go” to the university’s health service or other professionals, the belief that their distress would not be serious enough to warrant professional help was rated as the most important barrier, followed by the desire to handle the issue independently, and the belief it would cost too much money (Table 3). In contrast, anticipation of problems with time, transportation, and/or scheduling were rated to be the least important barriers. A small number of students also reported concerns that it would harm their career prospects. No significant differences were observed between genders or year level or either support avenue.

#### 3.2.3. Longitudinal Analyses

At Time 2, 17.13% of students said they would “definitely go” to the university’s health service if they developed a problem that caused emotional distress or interfered with their work, with a further 27.62% saying they “probably would go.” The distribution of responses did not differ over time *V* = 2936.5, *p* = 0.74. Additionally, 18.23% said they “definitely would go” somewhere else for help (e.g., GP), with a further 25.41% saying they “probably would go.” Again, the distribution of responses did not differ over time *V* = 3124, *p* = 0.90.

Perceived stigma did not change significantly over time, *V =* 4989, *p* = 0.68. There was little evidence for change over time for barriers to seeking help. The single exception was a significant change regarding time, scheduling and/or transport being a barrier to treatment, with reporting increasing from Time 1 (M = 2.24, SD = 1.20) to Time 2 (M = 2.41, SD = 1.23, *V =* 2999.5, *p* = 0.047).

### 3.3. Discussion

The current study aimed to understand the barriers students perceive to limit their ability to seek help when they experience mental distress and/or suicidal ideation. Relatively few students in the current study indicated that they would seek support for mental health distress, which is consistent with previous research [2]. In addition, students in this study endorsed similar perceptions of help-seeking barriers to previous research [2,15]. Specifically, the *desire to handle the issue independently* emerged as a strongly perceived barrier, following the perception that *students believed their issues as not serious enough to warrant help*. This is concerning, especially given the high number of participants whose scores indicated they were experiencing clinically significant depression, anxiety, and/or stress symptoms and/or recent thoughts about suicide.

One explanation for the consistent reporting of these barriers is that experiences of mental distress may be normalised in the student population, such that mental distress is viewed as part of being a student studying at university [16,17]. Alternatively, it may be that they continuously alter their definition of mental distress to accommodate their current state [18]. These findings indicate a need to increase mental health literacy and facilitate greater engagement with support services to enable earlier detection of, and intervention for, mental distress [19].

The only change observed over time was an increase in anticipating *greater problems with time, scheduling, and/or transport*. Unlike individual-level factors (e.g., how students perceive their problems), these practical challenges to seeking treatment are difficult to address and likely reflect increasing study demands and work commitments. This also highlights the possibility that students do not feel that they can make or find time to look after their mental health which may, in turn, elevate the risk of mental distress and/or suicidal ideation. It is important to note, however, that the wording of the statement lacks the necessary nuance to discriminate whether it is specifically time, scheduling, transport, or some combination thereof, that is the main issue.

Following on from these findings about individual-level factors, the aim of Study 2 was to assess the impact of a brief mental-health promotion video and infographic. The video and infographic specifically highlighted the main support services available to students, with the intention of increasing their intention to engage with health services and support.

## 4. Study 2

### 4.1. Method

#### 4.1.1. Participants

Participants were 133 undergraduate psychology students enrolled at a New Zealand University. Participants were between the ages of 18–35 years old (M = 20.17, SD = 2.31) and the majority identified as female (89.63%, *n* = 121, 11 males, one non-binary). Most participants identified as New Zealand European (78.52%, *n* = 106); the remaining participants identified as Māori (*n* = 6), Asian (*n* = 14), Pasifika (*n* = 2). Eight (6.02%) stated specific ethnicities which generally fell into other European regions. It is also important to note that participants could select multiple ethnicities.

#### 4.1.2. Procedure

**Figure 1:**
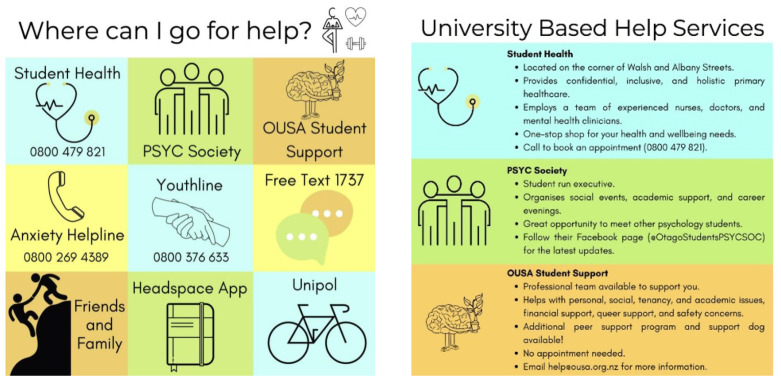
Examples of the infographic intervention. On the left, the initial 3 × 3 grid depicts all services and on the right the more detailed panel provides more information about the services.

This study was approved by the host university’s Ethics Committee (H21/022). Similar to Study 1, undergraduate psychology students were invited to participate for course credit. Students who volunteered to participate were randomly allocated to one of three groups: control, video, or infographic. Participants in the control group were given information about standard helplines only. Participants in the video group were shown a three-minute clip that included nine, thirty-second snippets detailing the locations and services of various university-based support services (e.g., the university’s health service), national helplines (e.g., 1737), and well-being-based help services (e.g., Headspace App). Participants in the infographic group were presented with written information corresponding to the same nine services described in the video. The names of the services were first presented in a 3 × 3 grid alongside a related image (see Figure 1). They were then further elaborated upon across three panels, with each panel describing three services.

All participants were asked to complete three electronic surveys using Qualtrics across a six-week period (Baseline, Week One; Post-Intervention, Week Three, Follow-Up, Week Seven). Each survey link, along with reminders, was emailed to participants. For participants in the intervention groups (infographic and video), the material was embedded into the Qualtrics survey (at the end of survey one and the start of survey two). All participants were given the infographic and video at the conclusion of the study.

#### 4.1.3. Measures

The same measures as Study 1 were used, with the exception of the PHQ-9 item related to suicidal ideation. Internal consistency was high across all scales: Depression (*Cronbach’s alpha* (*α*) = 0.893), Anxiety (*Cronbach’s alpha* (*α*) = 0.822), Stress (*Cronbach’s alpha * (*α*) = 0.796).

#### 4.1.4. Data Analysis

All analyses were conducted using R (version 4.2.1. [11]). First, descriptive statistics and frequencies were calculated (package: psych [12]). Kruskal–Wallis rank sum tests were used to understand whether group endorsements of perceived barriers changed over time or whether groups differed in the perceived barriers post-intervention (Time 2). Finally, linear mixed models were then used to determine whether any Group X Time interaction effects were present for each perceived help-seeking barrier (package: lmerTest [20]).

### 4.2. Results

#### 4.2.1. Clinical Characteristics

Using the clinical cut-off scores for the DASS-21 [14] at Time 1, 48.9% of the sample scored within the normal range for depression, 16.5% in the mild range, 21.8% in the moderate range, 8.3% in the severe range, and 4.5% in the extremely severe range. For anxiety, 37.6% of the sample scored within the normal range, 23.3% in the mild range, 17.3% in the moderate range, 7.5% in the severe range, and 14.3% in the extremely severe range. Additionally, 49.6% of the sample scored within the normal range for stress, 24.1% in the mild range, 18.0% in the moderate range, 6.8% in the severe range, and 1.5% in the extremely severe range. These are comparable to the sample used with Study 1.

#### 4.2.2. Help-Seeking Intentionality

No significant within-group changes in intentions for seeking help from the university’s health service or other professions were observed over time for any group (see Table 4). Likewise, no between-groups differences were observed post-intervention for intentions to seek support from the university’s health service. On the other hand, a significant difference between the control and intervention groups was observed post-intervention for students who would seek support from other services (*p* = 0.035). Participants in the infographic group were significantly more likely to seek support compared to the control group, M_Infographic_ = 3.52, SD = 1.02 vs. M_Control_ = 3.05, SD = 1.03; no differences were observed between the control and video groups or infographic and video groups, however.

#### 4.2.3. Reasons for Not Seeking Help

Across all ten help-seeking barriers, no significant within-group changes were observed over time for any group. Likewise, across the barriers, neither the infographic nor video groups significantly differed from the control group. A post-intervention difference was observed between the video and infographic groups regarding the perceived financial barrier to seeking help, with those in the infographic group being significantly more likely to believe that financial costs would limit their access to support, M_Infographic_ = 3.60, SD = 1.25 vs. M_Video_ = 2.94, SD = 1.26, *p* = 0.017.

Specifically looking at post-intervention (Time 2), one significant Group X Time interaction emerged. Participants in the infographic group were significantly more inclined to disagree that their distress was not serious enough to warrant support, B = −0.63, *p* = 0.009. This effect was not maintained over time, however. No other post-intervention interaction effects were observed (full results can be found within the Supplementary Materials).

### 4.3. Discussion

The current study found evidence that an infographic about available mental health services had a positive effect on intentions to seek help from mental health professionals outside the university’s health services but no evidence was found to support the effect of a short education-based video. These findings partially support previous promising evidence that interventions may be able to generate positive shifts in attitude towards seeking help when experiencing mental distress [5,6].

In considering why the infographic was more effective, one possibility is that the infographic allowed easier absorption of information by viewers than the video. It could subsequently be viewed more passively than a more time-demanding video [21]. While these results align with asthma-related research showing an infographic intervention is more effective than a video [21], they do differ from breast cancer screening research which found that videos are more effective [22]. As such, the effectiveness of an infographic compared to a video in communicating health-related information may depend on the target audience, the specific health issue addressed, and the design of the media. Future research should continue to explore what aspects of media best connect with university students to improve their intentions towards seeking support.

Perhaps more importantly, the Group X Time interaction for students’ belief that their issues are not serious enough to warrant help suggests that the infographic may have increased mental health literacy about when to seek help and potentially reduced comparisons between individuals (i.e., countering the effects observed in past literature [16,17]). This is critical from a prevention lens that focuses on intervening before the individual's distress escalates.

A limitation of the infographic intervention was that it only described the services and may not have sufficiently engaged students to enact a behavioural change [5]. While it may increase awareness about different services and when to seek help, more specific and concerted efforts may be required to increase engagement with support services. Furthermore, this study did not include a specific measure related to suicide. Although the DASS-21 Depression subscale has been found to predict suicide (e.g., Ibrahim et al. [23]), replication of this study with explicit measures for suicide is required.

## 5. General Conclusions

This series of studies has contributed to the emerging literature on university student’s attitudes and behaviours towards help-seeking by (1) replicating earlier cross-sectional work by the WHO World Mental Health Surveys International College Student Project [1,2], (2) longitudinally testing whether students’ perceptions and intentions to seek help if experiencing mental distress shift over time, and (3) examining whether a small-scale intervention can shift student’s attitudes and behaviours towards help-seeking if distress were to be experienced.

Consistent with prior research, the findings of the current studies have highlighted that, among Aotearoa New Zealand university students, there is a perception that their issues are not serious enough to warrant support and a desire to handle the issues independently. Concerningly, these perceptions were also paired with clinically relevant symptoms of depression and anxiety, and suicidal ideation. Furthermore, we also found increased perceptions of barriers to help-seeking related to time, scheduling, or transport preventing the ability for one to seek support. The combination of these findings presents a key paradox that, although informational interventions may be able to shift students’ attitudes and intentions towards seeking help, until methods are found to increase the capacity for them to engage with support services, efforts in this area may not result in the desired reductions in negative outcomes when mental distress is experienced (e.g., prevention of suicide, positive academic outcomes).

The overall tendency to disagree that seeking professional psychological help is associated with negative public stigma aligns with previous research that broader perceptions about what others may think do not appear to prevent students from seeking help [15,16,24]. It is important to note, however, that while overall perceptions of public stigma were low, males were still more likely to perceive greater levels of public stigma than females; a finding that is, perhaps, unsurprising given the hegemonic masculinity ideals (e.g., kiwi stoicism [25]) that remain entrenched within the Aotearoa New Zealand context. Although the overall reduction reflects a shift in this area, a continued focus on reducing stigma related to mens’ help-seeking is required.

This study is not without limitations. Beyond those already discussed, it is also essential to consider the barriers examined are not an exhaustive list of reasons that may reduce a student’s willingness to seek help. Although they were replicated from prior research [1], future research should extend this research to consider broader help-seeking barriers, for example, the fear of losing autonomy or involuntary hospitalisation [26].

Positively, the present study presents evidence that a simple infographic intervention can shift a student’s perspective that their distress is not serious enough to seek support. This could have real-world applications as it suggests that universities may be able to positively engage students through social media-style content and, as such, is a low-cost mechanism for intervention. Further efforts would be required to ensure that the effects of such interventions persist over time and translate into greater engagement with support services, provided support services are accessible. If effective, they could easily be adapted for specific purposes (e.g., suicide prevention, exam-related stress).

## 6. Conclusions

In conclusion, this series of studies contributes to our understanding of the barriers that may prevent university students from seeking help and ways that may help to shift their perceptions of these barriers. By addressing these perceived barriers, it may be possible to facilitate students towards earlier engagement with support services and subsequently reduce the risk of progression to severe mental distress and/or suicide. Future research should expand the proposed intervention method to examine its utility and effectiveness in different countries and populations, and whether it can be targeted for specific samples.

## Figures and Tables

**Table 1:** Spearman’s rho correlations between Suicidal Ideation, Stigma, Depression, Anxiety, and Stress, *and* barriers to help-seeking for Study One.

	R1	R2	R3	R4	R5	R6	R7	R8	R9	R10
**Time 1**										
Suicidal Ideation	−0.05	0.14 *	−0.05	0.05	−0.24 ***	0.06	−0.03	0.10 *	0.15 **	0.09
Depression	0.09	0.10 *	0.06	0.08	−0.30 ***	0.11 *	0.03	0.15 **	0.12 *	0.10 *
Anxiety	0.14 **	0.12 *	0.11 *	0.10 *	−0.26 ***	0.09	0.03	0.17 ***	0.20 ***	0.15 **
Stress	0.14 **	0.13 **	0.03	0.10 *	−0.22 ***	0.13 **	0.11 *	0.18 ***	0.20 ***	0.12 **
Stigma	0.06	0.11 *	0.09	0.24 ***	−0.15 **	0.05	−0.08	0.02	0.16 ***	0.31 ***
**Time 2**										
Suicidal Ideation	0.15 *	0.18 **	0.02	0.12	−0.21 **	0.11	0.05	0.11	0.25 ***	0.12
Depression	0.19 **	0.18 *	0.12	0.11	−0.32 ***	0.06	0.032	0.13	0.12 **	0.15 *
Anxiety	0.04	0.14	−0.03	−0.06	−0.22 **	0.16 *	−0.01	0.09	0.06	−0.01
Stress	0.20	0.17 *	0.07	−0.02	−0.21 **	0.07	0.05	0.16 *	0.20 **	0.09
Stigma	0.16*	0.27 ***	0.12	0.23 **	−0.13	0.09	0.09	0.08	0.24 ***	0.35 ***

*Note.* * *p* < 0.05, ** *p* < 0.01, *** *p* < 0.001. R1: You do not believe that your distress is/was not serious enough to warrant professional help; R2: You are not sure the available treatments are very effective; R3: You would want to handle the problem on your own; R4: You would be too embarrassed; R5: You would talk to friends or relatives instead; R6: You think it costs too much money; R7: You are unsure of where to go or who to see; R8: You anticipate problems with time, transportation, or scheduling; R9: You are afraid it might harm you school or professional career; R10: You would worry that people would treat you differently if they knew you were in treatment.

**Table 2:** Frequencies of student’s intentions to engage with support services if they experienced distress.

Option	The University’s Health Service (%)	Other Professionals (%)
Definitely would not go	16 (4.28)	12 (3.21)
Probably would not go	55 (14.71)	73 (19.52)
Might or might not go	122 (32.62)	116 (31.02)
Probably would go	124 (33.16)	117 (31.28)
Definitely would go	56 (14.97)	55 (14.71)

**Table 3:** Perceived barriers to seeking help at Time 1 by gender and year.

Reason	Mean (SD)				
	Overall	Men	Women	Year One	Year Two
You do not believe that your distress is/was not serious enough to warrant professional help.	3.27 (1.15)	3.22 (1.09)	3.29 (1.16)	3.27 (1.14)	3.28 (1.17)
You would want to handle the problem on your own.	3.23 (1.20)	3.24 (1.40)	3.23 (1.16)	3.16 (1.17)	3.33 (1.24)
You think it costs too much money.	3.07 (1.38)	2.74 (1.39)	3.13 (1.37)	3.10 (1.39)	3.02 (1.36)
You would talk to friends or relatives instead.	3.02 (1.27)	3.04 (1.41)	3.02 (1.24)	2.99 (1.28)	3.07 (1.26)
You are unsure of where to go or who to see.	2.74 (1.27)	2.63 (1.17)	2.76 (1.29)	2.79 (1.29)	2.67 (1.24)
You would be too embarrassed.	2.56 (1.29)	2.69 (1.33)	2.54 (1.29)	2.55 (1.35)	2.59 (1.22)
You are not sure the available treatments are very effective.	2.55 (1.12)	2.74 (1.14)	2.51 (1.11)	2.58 (1.12)	2.51 (1.12)
You would worry that people would treat you differently if they knew you were in treatment.	2.50 (1.27)	2.76 (1.34)	2.45 (1.25)	2.49 (1.33)	2.52 (1.20)
You are afraid it might harm you school or professional career.	2.22 (1.32)	2.26 (1.46)	2.22 (1.29)	2.13 (1.27)	2.35 (1.37)
You anticipate problems with time, transportation, or scheduling.	2.20 (1.18)	2.06 (1.28)	2.23 (1.16)	2.25 (1.20)	2.13 (1.16)

**Table 4:** Mean and standard deviation of intention to seek help and the perceived barriers to seeking help by intervention group and time.

Group	Time	*n*	Mean (SD)											
			The University’s Health Service	Other Professional	R1	R2	R3	R4	R5	R6	R7	R8	R9	R10
C	T1	44	3.16 (1.26)	3.34 (1.03)	3.07 (1.02)	2.50 (1.15)	3.43 (1.00)	2.70 (1.29)	3.05 (1.18)	3.14 (1.27)	3.07 (1.19)	2.57 (1.28)	2.20 (1.15)	2.57 (1.19)
C	T2	44	3.09 (1.16)	3.05 (1.03)	3.27 (0.79)	2.59 (1.04)	3.36 (0.92)	2.59 (1.13)	2.98 (1.11)	3.07 (1.28)	2.68 (1.22)	2.45 (1.07)	2.25 (1.28)	2.32 (1.07)
C	T3	44	3.25 (1.14)	3.18 (1.04)	3.02 (1.02)	2.57 (1.19)	3.20 (1.02)	2.32 (1.09)	2.95 (1.08)	2.93 (1.19)	2.75 (1.18)	2.52 (1.23)	2.30 (1.30)	2.43 (1.07)
I	T1	42	3.67 (1.05)	3.52 (1.02)	3.40 (1.25)	2.62 (1.15)	2.98 (1.07)	2.21 (1.20)	3.14 (1.12)	3.50 (1.42)	2.93 (1.26)	2.52 (1.25)	2.24 (1.30)	2.26 (1.29)
I	T2	42	3.40 (1.11)	3.52 (1.02)	2.98 (1.12)	2.57 (1.15)	3.02 (1.16)	2.19 (0.89)	3.12 (1.13)	3.60 (1.25)	2.62 (1.17)	2.60 (1.23)	2.26 (1.13)	2.12 (1.15)
I	T3	42	3.31 (1.05)	3.36 (1.1)	3.26 (1.13)	2.57 (1.06)	3.17 (1.10)	2.26 (1.13)	3.02 (1.05)	3.29 (1.17)	2.52 (1.11)	2.57 (1.13)	2.40 (1.29)	2.26 (0.94)
V	T1	47	3.36 (1.19)	3.32 (1.14)	3.21 (1.16)	2.47 (1.00)	3.21 (1.20)	2.34 (1.11)	3.17 (1.24)	3.32 (1.30)	3.00 (1.23)	2.38 (1.23)	1.94 (1.13)	2.00 (1.22)
V	T2	47	3.09 (1.16)	3.26 (1.19)	3.30 (1.00)	2.36 (0.94)	3.32 (1.14)	2.26 (1.28)	3.00 (1.16)	2.94 (1.26)	2.62 (1.07)	2.45 (1.16)	2.00 (1.04)	1.91 (1.04)
V	T3	47	3.26 (1.07)	3.23 (1.13)	3.45 (1.00)	2.60 (1.06)	3.28 (1.04)	2.23 (1.24)	3.28 (0.93)	3.15 (1.18)	2.72 (1.21)	2.60 (1.19)	2.09 (1.21)	2.09 (1.16)

R1: You do not believe that your distress is/was not serious enough to warrant professional help; R2: You are not sure the available treatments are very effective; R3: You would want to handle the problem on your own; R4: You would be too embarrassed; R5: You would talk to friends or relatives instead; R6: You think it costs too much money; R7: You are unsure of where to go or who to see; R8: You anticipate problems with time, transportation, or scheduling; R9: You are afraid it might harm you school or professional career; R10: You would worry that people would treat you differently if they knew you were in treatment.

## Data Availability

The data presented in this study are available on request from the corresponding author.

## Supplementary Materials

The following supporting information can be downloaded at: https://www.mdpi.com/article/10.3390/ijerph192315836/s1, Table S1: Spearman’s rho Intercorrelations between Suicidal Ideation, Stigma, and symptoms of Depression, Anxiety, and Stress at Time One, Study One; Table S2: Linear Mixed Models examining Group X Time interactions for each help-seeking barrier.
