# Supplementary material for: Understanding Aotearoa New Zealand University Students Intentions to Seek Help If Experiencing Mental Distress: A Comparison of Naturalistic and Interventional Findings
Source: Int J Environ Res Public Health. 2022 Nov 28;19(23):15836. doi: 10.3390/ijerph192315836 (PMC9738961; doi:10.3390/ijerph192315836)
Supplement: Supplementary file 1 [file ijerph-19-15836-s001.zip › ijerph-2013268-supplementary.pdf]

Supplementary Analyses

Table S1. Spearman's rho Intercorrelations between Suicidal Ideation, Stigma, and symptoms of Depression, Anxiety, and Stress at Time One, Study One.

	Depression		Anxiety		Stress		Stigma	
Depression	—							
Anxiety	0.575	***	—					
Stress	0.686	***	0.694	***	—			
Stigma	0.198	***	0.211	***	0.174	***	—	
Suicidal Ideation	0.437	***	0.367	***	0.316	***	0.169	***

International Journal of
Environmental Research and Public Health

MDPI

Table S2. Linear Mixed Models examining Group X Time interactions for each help-seeking barrier.

	R1			R2			R3			R4			R5		
**Predictors**	**Estimate**	**CI**	**p**	**Estimate**	**CI**	**zz**	**Estimate**	**CI**	**p**	**Estimate**	**CI**	**p**	**Estimate**	**CI**	**p**
(Intercept)	3.07	2.75 – 3.38	<0.001	2.5	2.18 – 2.82	<0.001	3.43	3.11 – 3.75	<0.001	2.7	2.36 – 3.05	<0.001	3.05	2.72 – 3.38	<0.001
Group [I]	0.34	-0.11 – 0.79	0.141	0.12	-0.34 – 0.58	0.61	-0.46	-0.91 – 0.00	0.05	-0.49	-0.98 – 0.00	0.05	0.1	-0.37 – 0.57	0.685
Group [V]	0.14	-0.29 – 0.58	0.515	-0.03	-0.48 – 0.41	0.888	-0.22	-0.66 – 0.22	0.332	-0.36	-0.84 – 0.11	0.135	0.12	-0.33 – 0.58	0.593
Time [T2]	0.2	-0.13 – 0.54	0.227	0.09	-0.23 – 0.41	0.576	-0.07	-0.39 – 0.25	0.674	-0.11	-0.41 – 0.19	0.458	-0.07	-0.38 – 0.24	0.664
Time [T3]	-0.05	-0.38 – 0.29	0.788	0.07	-0.25 – 0.39	0.675	-0.23	-0.55 – 0.09	0.162	-0.39	-0.69 – -0.09	0.012	-0.09	-0.40 – 0.22	0.562
Group [I] * Time [T2]	-0.63	-1.11 – -0.16	0.009	-0.14	-0.60 – 0.32	0.552	0.12	-0.34 – 0.57	0.618	0.09	-0.34 – 0.52	0.682	0.04	-0.40 – 0.48	0.843
Group [V] * Time [T2]	-0.12	-0.58 – 0.34	0.612	-0.2	-0.64 – 0.25	0.384	0.17	-0.27 – 0.62	0.44	0.03	-0.39 – 0.45	0.894	-0.1	-0.53 – 0.33	0.64
Group [I] * Time [T3]	-0.1	-0.57 – 0.38	0.688	-0.12	-0.57 – 0.34	0.619	0.42	-0.04 – 0.87	0.073	0.43	0.00 – 0.86	0.048	-0.03	-0.47 – 0.41	0.9
Group [V] * Time [T3]	0.28	-0.18 – 0.74	0.236	0.06	-0.39 – 0.50	0.793	0.29	-0.15 – 0.73	0.198	0.28	-0.14 – 0.70	0.19	0.2	-0.23 – 0.63	0.366
	Random Effects														
σ2			0.63			0.63			0.63			0.63			0.63
τ00			0.49 StudentID			0.49 StudentID			0.49 StudentID			0.49 StudentID			0.49 StudentID
ICC			0.44			0.44			0.44			0.44			0.44
N			133 StudentID			133 StudentID			133 StudentID			133 StudentID			133 StudentID
Observations			399			399			399			399			399
Marginal R2 / Conditional R2			0.021 / 0.450			0.021 / 0.450			0.021 / 0.450			0.021 / 0.450			0.021 / 0.450

	R6			R7			R8			R9			R10		
**Predictors**	**Estimates**	**CI**	**p**	**Estimates**	**CI**	**p**	**Estimates**	**CI**	**p**	**Estimates**	**CI**	**p**	**Estimates**	**CI**	**p**
(Intercept)	3.14	2.76 – 3.51	<0.001	3.07	2.72 – 3.42	<0.001	2.57	2.21 – 2.92	<0.001	2.2	1.85 – 2.56	<0.001	2.57	2.23 – 2.90	<0.001
Group [I]	0.36	-0.17 – 0.90	0.182	-0.14	-0.64 – 0.36	0.585	-0.04	-0.55 – 0.46	0.864	0.03	-0.48 – 0.54	0.897	-0.31	-0.79 – 0.17	0.209
Group [V]	0.18	-0.34 – 0.70	0.49	-0.07	-0.56 – 0.42	0.784	-0.19	-0.68 – 0.31	0.462	-0.27	-0.77 – 0.23	0.289	-0.57	-1.03 – -0.10	0.017

Time [T2]	-0.07	-0.39 – 0.25	0.676	-0.39	-0.72 – -0.05	0.025	-0.11	-0.45 – 0.22	0.505	0.05	-0.28 – 0.37	0.785	-0.25	-0.55 – 0.05	0.102
Time [T3]	-0.2	-0.53 – 0.12	0.211	-0.32	-0.66 – 0.02	0.065	-0.05	-0.38 – 0.29	0.79	0.09	-0.24 – 0.42	0.585	-0.14	-0.44 – 0.16	0.372
Group [I] * Time [T2]	0.16	-0.30 – 0.62	0.484	0.08	-0.41 – 0.56	0.755	0.19	-0.29 – 0.66	0.448	-0.02	-0.49 – 0.45	0.928	0.11	-0.32 – 0.54	0.624
Group [V] * Time [T2]	-0.31	-0.76 – 0.13	0.166	0	-0.47 – 0.47	0.989	0.18	-0.29 – 0.64	0.454	0.02	-0.44 – 0.47	0.937	0.16	-0.25 – 0.58	0.438
Group [I] * Time [T3]	-0.01	-0.47 – 0.45	0.967	-0.09	-0.57 – 0.40	0.725	0.09	-0.39 – 0.57	0.703	0.08	-0.39 – 0.54	0.75	0.14	-0.29 – 0.57	0.533
Group [V] * Time [T3]	0.03	-0.41 – 0.48	0.88	0.04	-0.43 – 0.51	0.862	0.26	-0.21 – 0.72	0.276	0.06	-0.40 – 0.51	0.802	0.22	-0.20 – 0.64	0.298
Random Effects															
σ2			0.63			0.63			0.63			0.63			0.63
τ00			0.49 StudentID			0.49 StudentID			0.49 StudentID			0.49 StudentID			0.49 StudentID
ICC			0.44			0.44			0.44			0.44			0.44
N			133 StudentID			133 StudentID			133 StudentID			133 StudentID			133 StudentID
Observations			399			399			399			399			399
Marginal R2 / Conditional R2			0.021 / 0.450			0.021 / 0.450			0.021 / 0.450			0.021 / 0.450			0.021 / 0.450

R1: You do not believe that your distress is/was not serious enough to warrant professional help; R2: You are not sure the available treatments are very effective; R3: You would want to handle the problem on your own; R4: You would be too embarrassed ; R5: You would talk to friends or relatives instead ; R6: You think it costs too much money ; R7: You are unsure of where to go or who to see ; R8: You anticipate problems with time, transportation, or scheduling ; R9: You are afraid it might harm you school or professional career ; R10: You would worry that people would treat you differently if they knew were in treatment.
